# Breaking Barriers in Cardiovascular and Cancer Care With Multimodal AI

**DOI:** 10.1016/j.jacadv.2024.101433

**Published:** 2024-12-10

**Authors:** Christopher L.F. Sun

**Affiliations:** a Telfer School of Management, University of Ottawa, Ottawa, Ontario, Canada; b University of Ottawa Heart Institute, University of Ottawa, Ottawa, Ontario, Canada

**Keywords:** artificial intelligence, cancer, cardiovascular events, immune checkpoint inhibitor, myocarditis

**Figure:**
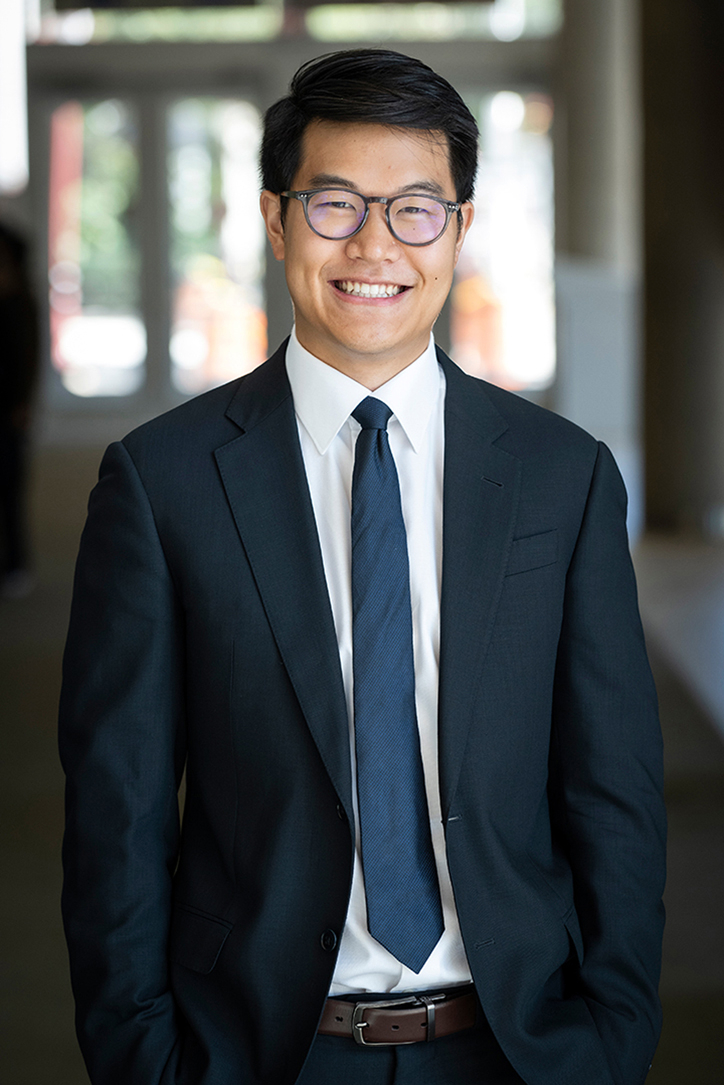

Immune checkpoint inhibitors (ICIs) have dramatically shifted the landscape of cancer treatment, offering new hope for patients by harnessing the immune system.^1^ However, along with these benefits comes cardiovascular complications, including myocarditis, pericarditis, arrhythmias, and other vascular events.^2^^,^^3^ These conditions carry a risk of morbidity and mortality and their onset can range from weeks to years after initiation of therapy, complicating the clinical management of patients undergoing ICI therapy.^4^

The identification of patients at elevated risk for these cardiovascular events is vital to delivering effective and personalized care. Currently, diagnostic pathways for ICI-related myocarditis, including invasive biopsy and advanced imaging, like cardiac magnetic resonance imaging, are resource-intensive and often not suitable for severely ill patients.^5^^,^^6^ There remains a pressing need for accessible, noninvasive predictive models to stratify risk before or early in the treatment course.

In this issue of *JACC: Advances*, Ayoub et al^7^ developed and presented a novel multimodal deep learning model to predict the risk of a composite endpoint of ICI-related myocarditis and major adverse cardiovascular events in cancer patients. The study included 2,258 patients receiving ICI therapy at 3 academic institutions in the United States, of whom 11.7% experienced cardiovascular adverse events (with 3% developing myocarditis). The multimodal predictive model integrates both electrocardiogram (ECG) data and electronic medical record data (eg, demographics, comorbidities, and laboratory values), outperforming models using either modality alone. The model achieved an out-of-sample area under the receiver operating characteristic curve (AUC) of 0.717, with a true positive rate of 64.28% and a negative predictive value of 0.98 after optimal binary prediction threshold selection.

Beyond its importance toward improving ICI treatment planning, the study provides valuable insights on predictive modeling in cardiology and health care for multiple reasons. Firstly, the integration of AI models in predicting cardiovascular events among cancer patients receiving ICIs represents a transformative approach that transcends traditional disease boundaries. These models allow for the early identification of patients at risk of cardiotoxicity, enabling personalized strategies that reduce cardiovascular complications without compromising cancer treatment efficacy. This holistic approach to patient care breaks down traditional silos between specialties, enhancing patient outcomes and deepening our understanding of the interplay between cancer therapies and cardiovascular health.

Secondly, this study underscores the utility of multimodal predictive models, which integrate diverse data types to emulate human’s ability to process and synthesize information from various sources, leading to more comprehensive and accurate predictions.^8^^,^^9^ Ayoub et al convincingly demonstrate the benefits of combining clinical data with ECG readings (AUC = 0.717) over single-modality approaches (AUC = 0.59-0.645), significantly improving prediction accuracy. Interestingly, the inclusion of echocardiography reports, another modality, did not enhance the model's performance (AUC = 0.670) for this particular prediction task. The authors suggest that this counterintuitive finding could stem from the inconsistent availability of echocardiography data and predominance of normal results within this cohort. It emphasizes that the performance of predictive models is highly dependent on the quality, completeness, and relevance of input data. Further investigation into the use of rich data sources, particularly echocardiogram data in both image form and structured reports, is warranted to enhance the prediction of cardiac complications.

Third, the challenge of predicting rare events in imbalanced data sets remains a significant limitation in the advancement of predictive models in health care.^10^ In this study, Ayoub et al overcome this issue by utilizing a composite endpoint, combining ICI-related myocarditis with major adverse cardiovascular events. This pragmatic approach improves the feasibility of the model and enhances predictive performance by increasing the event rate. However, as highlighted by the authors, the rarity of individual events like myocarditis and the variability in event rates necessitate careful interpretation of the model's results. Indeed, this strategy introduces trade-offs as while the model can provide general predictions of adverse outcomes, its ability to inform targeted interventions for individual patients and specific cardiac events is reduced. This highlights a pressing need to collaborate and curate larger data sets to capture more instances of rare events, improving model precision and enabling more specific, actionable predictions. In the interim employing existing techniques to overcome the challenge of model development on imbalanced data sets, such as oversampling,^10^ could enhance model performance.

The multimodal AI model presented by Ayoub et al offers a crucial step forward in predicting ICI-associated cardiovascular risks, merging oncology and cardiology for a more integrated patient care approach. By combining ECG and electronic medical record data, it enhances early identification of high-risk patients, enabling personalized management strategies that minimize cardiotoxicity without interrupting cancer treatment. While challenges persist for the medical community, particularly in addressing data quality, data availability, and the rarity of events, these obstacles present opportunities for collaboration and innovation. By working together to improve data sharing and integrate multimodal data from diverse sources, we can refine predictive precision and develop more targeted, actionable interventions for at-risk patients. Building upon the foundation laid by this study, continued efforts will further advance precision medicine and improve outcomes for patients undergoing ICI therapy.

## Funding support and author disclosures

The author has reported that they have no relationships relevant to the contents of this paper to disclose.

## References

[1] Robert C. (2020). A decade of immune-checkpoint inhibitors in cancer therapy. Nat Commun.

[2] Lyon A.R., Yousaf N., Battisti N.M.L., Moslehi J., Larkin J. (2018). Immune checkpoint inhibitors and cardiovascular toxicity. Lancet Oncol.

[3] Thuny F., Naidoo J., Neilan T.G. (2022). Cardiovascular complications of immune checkpoint inhibitors for cancer. Eur Heart J.

[4] Curigliano G., Lenihan D., Fradley M. (2020). Management of cardiac disease in cancer patients throughout oncological treatment: ESMO consensus recommendations. Ann Oncol.

[5] Anon Defining cardiovascular toxicities of cancer therapies: an International cardio-oncology society (IC-OS) consensus statement | European heart journal | Oxford academic. https://academic.oup.com/eurheartj/article/43/4/280/6460956.

[6] Wang C., Zhao G., Zhang Z. (2023). Immune checkpoint inhibitor–associated myocarditis: a systematic analysis of case reports. Front Immunol.

[7] Ayoub, Chadi, Appari, Lalith, Pereyra, Milagros, et al. Multimodal Fusion Artificial Intelligence Model to Predict Risk for Cardiovascular Adverse Events and Myocarditis in Cancer Patients Receiving Immune Checkpoint Inhibitor Therapy. JACC Adv. 10.1016/j.jacadv.2024.101435 PMC11699614 39759436

[8] Acosta J.N., Falcone G.J., Rajpurkar P., Topol E.J. (2022). Multimodal biomedical AI. Nat Med.

[9] Kline A., Wang H., Li Y. (2022). Multimodal machine learning in precision health: a scoping review. NPJ Digit Med.

[10] Megahed F.M., Chen Y.-J., Megahed A., Ong Y., Altman N., Krzywinski M. (2021). The class imbalance problem. Nat Methods.

